# Effects of Black Soldier Fly Larvae (*Hermetia illucens* Larvae) Meal on the Production Performance and Cecal Microbiota of Hens

**DOI:** 10.3390/vetsci10050364

**Published:** 2023-05-19

**Authors:** Yan Yan, Jinjin Zhang, Xiaochen Chen, Zhanbin Wang

**Affiliations:** Henan Provincial Academician Workstation of Feed Resource Development and Healthy Livestock, Department of Animal Science and Technology, Henan University of Science and Technology, Luoyang 271023, China; yanyan@stu.haust.edu.cn (Y.Y.); chenxiaochen0844@126.com (X.C.)

**Keywords:** *Hermetia illucens*, larvae meal, production performance, laying hen, cecum microflo

## Abstract

**Simple Summary:**

The production performance of hen generally decreases with age, thereby reducing the forming profits of poultry farmers. Therefore, keeping egg production at a high rate is important. Gut microbial communities play a vital role in the health and function of the host. Animal gut microbiota are a complicated and diverse system easily affected by diet. Diet components could influence the composition and diversity of gut microbiota. In this study, hermetia illucens larva meal (HILM) was added to diets of hens to determine the effects of HILM on the production performance and cecal microbiota of hens in the late laying period. The results suggest that dietary supplementation with HILM had a significant effect on the production performance and cecal microflora of laying hens during the late laying period. It improved the laying rate, reduced the cracked egg rate, and improved the community richness and community diversity of the cecal microbial. Dietary supplementation with HILM had no adverse effect on the intestinal dominant flora.

**Abstract:**

The effects of *Hermetia illucens* larvae meal (HILM) as a feed supplement on production performance and cecal microflora were studied in 900 Hy-line Brown laying hens. Laying hens (60 weeks old) were randomly divided into four groups. Each group had five replicates, and each replicate had 45 hens. The control group was fed with a corn–soybean-based diet, and the experimental groups were fed with 1% HILM, 2% HILM, or 3% HILM. Results were as follows: (1) With the increase in HILM level, the laying rate increased linearly (*p* ≤ 0.05), and the feed/egg and cracked-egg rate decreased linearly (*p* ≤ 0.05). (2) Community composition analysis showed that the dominant bacteria in each group were *Bacteroidetes* and *Firmicutes*, followed by *Actinobacteria* and *Proteobacteria*, which accounted for more than 97% of 16S rRNA gene sequence of the total cecal bacteria. (3) Alpha diversity analysis at the operational taxonomic unit classification level showed that the HILM-addition groups had higher community richness and community diversity than the control group. (4) Principal co-ordinates analysis showed that the cecum samples in each group were significantly separated (*p* ≤ 0.05). At the phylum level, the relative abundance of *Bacteroidetes* in the HILM addition groups was significantly lower than that in the control group (*p* < 0.001), and the relative abundance of *Firmicutes* in the HILM addition groups was significantly higher than that in the control group (*p* < 0.001). In conclusion, dietary HILM supplementation had a significant effect on the production performance and cecal microflora of laying hens at the late laying period under the conditions of this experiment but had no adverse effect on the intestinal dominant flora.

## 1. Introduction

Insects are a huge and treasured resource and can be used as sources of human food and animal feed [1,2]. *Hermetia illucens* (*H. Illucens*) (Diptera: Stratiomyidae) is one of the candidate species with the most potential. *H. illucens* is a saprophytic insect [3], which has the characteristics of rapid reproduction, a high conversion rate, high nutritional value and easy feeding [4]. Its larvae can convert a variety of organic materials into a nutrient-rich biomass in animal feed [5]. Therefore, it can be used for the disposal of animal manure [5], restaurant waste [6], aquaculture organic waste [7], food-processing-plant byproducts, slaughterhouse wastes and waste fruits and vegetables [8]. *H. illucens* larvae contain a high concentration of protein and fat [9], as well as rich essential and nonessential amino acids [5,10]. The amounts of these essential amino acids in *H. illucens* larvae seem to be sufficient to meet the requirements of poultry production [5,11]. Therefore, in recent years, *H. illucens* larvae have been regarded as a valuable protein feed resource, used as ingredients in animal feed and human food [12] and incorporated into biodiesel production [13]. Among these, the most widespread use is in animal feed.

*H. illucens* larvae have been used as ingredients in animal feed for various animals, such as aquaculture [14,15], quails [16,17,18], barbary [19,20], muscovy ducks [21,22], broiler [23,24,25], turkey [26], and laying hens [27,28,29,30,31], pig [9,32,33,34,35], and rabbit [36,37].Various applications of *H. illucens* larvae in animal feed have achieved satisfactory results. Maurer et al. (2016) [38] showed that replacing 50% and 100% soybean meal of laying hens with partially defatted *Hermetia illucens* larvae meal (HILM) had no significant effect on the laying rate, egg weight, feed intake and feed conversion ratio. Yu Miao et al. (2019) [34] pointed out that HILM in the feed can promote the digestion and absorption of nutrients. In the previous studies, *H. illucens* larvae in animal feed have shown no adverse effects on performance and livestock product quality, and can strengthen immunity [39,40,41], change the intestinal microflora (increase bacterial diversity, especially the dominant flora), and improve intestinal health [9,34,42,43,44]. Animal intestinal microbiota are a complicated and diverse system that play a critical role in the health and function of the host. They are susceptible to many factors, such as age, diet, environment and hygiene level [45]. Dietary differences are the main cause of the total variations [46], indicating that diet components could affect the composition and diversity of intestinal microbiota.

Owing to the development of the diversified consumption of livestock products, livestock products have become increasingly popular. However, the production of eggs will decline with increasing age, which reduces the profits of farmers. Therefore, we tried to add HILM to the diet of laying hens to explore its influence on the production performance and cecal microbiota of laying hens, to maintain a high egg production rate. The late laying period is the key period for the performance of laying hens. Reports on the effects of *H. illucens* larvae on the intestinal microflora of laying hens in the late laying period are few; more research data on the use of insects are needed in animal feed assessment, and further reports on safety issues [47] are needed to further support the application of *H. illucens* in poultry diet. Therefore, this experiment was conducted to determine the effects of dietary supplementation with *H. illucens* larvae meal (HILM) on the production performance and cecal microbiota of Hy-line Brown laying hens to provide a novel and valuable reference for the application of HILM as a feed additive.

## 2. Materials and Methods

All the animal experimentation procedures were approved by the Institutional Animal Care and Use Committee of the Henan University of Science and Technology, Luoyang, China (HAUST-EAW-2021-C0401). The approval date is 16 February 2021.

### 2.1. HILM

HILM was provided by Zhengzhou Bennong Agricultural Technology Co., Ltd. (Zhengzhou, China). The main composition of the substrate used for *H. illucens* larvae feeding was chicken manure. Dry matter (DM), crude protein (CP), ether extract (EE), crude ash (ash), methionine (Met), lysine (Lys), calcium (Ca) and phosphorus (P) in HILM were determined by the method of AOAC (1999). Table 1 shows the chemical components of HILM.

### 2.2. Laying Hens, Diets, and Experimental Design

A total of 900 Hy-Line Brown laying hens (60 weeks old) were randomly divided into four groups (5 replicates in each group, and 45 hens in each replicate). The average laying rate and egg weights were 90.12 ± 0.28% and 68.12 ± 0.46 g (mean ± SEM). The differences in laying rate among the four groups before the start of the experiment were insignificant. The hens were raised in 300 wire cages, and 3 hens were raised in a cage (64 cm × 35 cm × 35 cm; length × width × height). The hens had free access to feed and water during the experiment and were exposed to a 16 h:8 h light/dark cycle. The average room temperature was 20 ± 3 °C. The whole process included a 7-day pre-experiment and 60-day formal experiment.

The groups were subjected to different dietary treatments. The control group was fed a corn–soybean meal-based diet, and the diets of the three treated groups were supplemented with 1%, 2%, and 3% HILM, respectively. The diets of the experimental groups were isonitrogenous and isoenergetic. According to the Management Guide of National Research Council [48], the diets were formulated to meet the nutrient requirements of laying hens. Table 2 shows the composition and nutrient levels of the diets. Crude protein (method 976.06), available phosphorus (method 993.31), calcium (method 927.02) and amino acid composition (method 994.12) were analysed in accordance with the method of AOAC (1999).

### 2.3. Production Performance

Egg production, cracked eggs, egg weight, and hen mortality were recorded daily during the experimental period. Feed consumption was recorded once a week. Laying rate (including cracked eggs), cracked egg rate, average egg weight, average daily feed intake (ADFI) and feed/egg ratio were analyzed. The laying rate and cracked eggs rate were calculated in percentages according to the formula: laying rate = total eggs/the number of hens × 100, cracked eggs rate = cracked eggs/total eggs × 100. Average egg weight was shown as the egg production weight per laying hen per day. ADFI was shown as the feed consumption weight per laying hen per day. Feed/egg ratio was the weight of the feed consumed when producing per unit egg weight. This was calculated by dividing the weight of feed consumed by the weight of eggs produced.

### 2.4. Cecal Microbiome

At the end of this experiment, five hens were randomly selected and euthanized by cervical dislocation from each group (one for each replication), and the cecum chyme samples were aseptically collected in 2 mL sterile frozen tubes (20 cecum chyme samples in total) and immediately stored at −80 °C for further analysis.

#### 2.4.1. Total Genomic DNA Extraction

Total genomic DNA was extracted from frozen cecal chyme samples with an E.Z.N.A^®^ soil DNA kit (Omega Bio-tek, Norcross, GA, USA) according to the manufacturer’s protocol.

#### 2.4.2. Quantify DNA Concentration

Final DNA concentration and purification were quantified with a NanoDrop 2000 spectrophotometer (Thermo Scientific, Wilmington, DE, USA), and DNA quality was detected through 1% agarose gel electrophoresis (Voltage: 5 V/cm, time: 20 min).

#### 2.4.3. PCR Amplification

The V3–V4 hypervariable regions of the bacterial 16S rRNA gene were amplified with primers 338F (5′-ACTCCTACGGGAGGCAGCAG-3′) and 806R (5′-GGACTACNNGGGTATCTAAT-3′) on a thermocycler PCR system (GeneAmp 9700, Foster City, ABI, CA, USA). PCR reactions were conducted as follows: denaturation at 95 °C for 5 min, followed by 27 cycles at 95 °C for 30 s, 55 °C for 30 s, and 72 °C for 45 s and a final extension at 72 °C for 10 min. They were performed in triplicate with 20 μL of mixture containing 4 μL of 5 × FastPfu buffer, 2 μL of 2.5 mM dNTPs, 0.8 μL of each primer (5 μM), 0.4 μL of FastPfu Polymerase and 10 ng of template DNA. All PCR products were purified using an AxyPrep DNA Gel Extraction Kit (Axygen Biosciences, Union City, CA, USA) and quantified using QuantiFluor™-ST (Promega, Madison, WI, USA) according to the manufacturer’s instructions.

#### 2.4.4. Illumina MiSeq Sequencing

Purified amplicons were pooled in equimolar concentrations from each sample and paired-end sequenced (2 × 300) on an Illumina MiSeq platform (Illumina, San Diego, CA, USA) according to the standard protocols of Majorbio Bio-Pharm Technology Co., Ltd. (Shanghai, China). The raw reads were deposited into the NCBI Sequence Read Archive database (accession PRJNA746625).

#### 2.4.5. Processing of Sequencing Data

Raw sequence data generated from 16S rRNA MiSeq sequencing were demultiplexed, quality-filtered using Trimmomatic and merged using FLASH with the following criteria: (i) the reads were truncated at any site receiving an average quality score < 20 over a 50-base pair (bp) sliding window; (ii) the primers were exactly matched, thereby allowing for two-nucleotide mismatching, and the reads containing ambiguous bases were removed; and (iii) sequences with overlaps longer than 10 bp were merged on the basis of their overlap sequences.

Operational taxonomic units (OTUs) were clustered with a 97% similarity cut-off with UPARSE (version 7.1 http://drive5.com/uparse/, accessed on 18 September 2021), and chimeric sequences were identified and removed using UCHIME. The taxonomy of each 16S rRNA gene sequence was analysed using the RDP classifier algorithm (version 2.11 http://rdp.cme.msu.edu/, accessed on 18 September 2021) against the Silva (SSU123) 16S rRNA database with a confidence threshold of 70%.

### 2.5. Statistical Analysis

A total of 900 laying hens were divided into four groups (5 replicates in each group, and 45 hens in each replicate). Data were analyzed with one-way ANOVA, and SPSS 22.0 (SPSS Inc., Chicago, IL, USA) was used. The model for one-way ANOVA was:

Y_ij_ = µ + D_i_ + e_ij_

with Y as the single observation; µ = overall mean; D_i_ = effect of diet (i = 0, 1, 2, 3); e_ij_ = effect of the error;

In addition, linear or quadratic responses were also analyzed by SPSS 22.0. Data significance was determined at *p* ≤ 0.05.

Rarefaction curve and alpha diversity analyses consisting of community richness (Sobs, Chao and Ace indices), community diversity (Simpson and Shannon indices), and community coverage (coverage indices) were performed using mothur (version v.1.30.1) on the basis of the summary single command for the observation of sampling efficiency and diversity. The distribution proportions of dominant species in different groups at the phylum, family, and genus levels were determined using Circos. Diversity among samples was investigated through beta diversity analysis. Principal co-ordinates’ analysis (PCoA) was calculated by the R package to describe the distances among samples, and ANOSIM was used to conduct statistically significant analyses at the the phylum, family, and genus levels.

## 3. Results

### 3.1. Production Performance

The effects of HILM on the production performance of laying hens are shown in Table 3. There were linear increases in laying rate as the amounts of HILM increased (*p* ≤ 0.05), with a higher laying rate for hens fed 2% HILM and 3% HILM than control hens (*p* ≤ 0.05). Linear decreases in feed/egg were present as the amounts of HILM increased (*p* ≤ 0.05), with a lower feed/egg rate for hens fed 2% HILM and 3% HILM than control hens (*p* ≤ 0.05). There were linear decreases in cracked-egg rate as the amounts of HILM increased (*p* ≤ 0.05), with a lower cracked-egg rate for hens fed 3% HILM than control hens (*p* ≤ 0.05).

### 3.2. Cecal Microbiota

The microbial composition of the cecum chyme in the laying hens subjected to HILM treatment was revealed through sequencing with 16S rRNA Illumina Miseq. A total of 1,049,600 V3-V4 16S rRNA effective sequences were obtained from 20 samples and used for subsequent analysis. An average of 52,480 sequences were obtained per sample, with the minimum number (35,065) and maximum number (74,424).

#### 3.2.1. Rarefaction Curves

Rarefaction curves generated from the OTU showed that the sampling in each group Provided sufficient sampling coverage (Figure S1).

#### 3.2.2. Alpha Diversity Analysis

**Figure 1:**
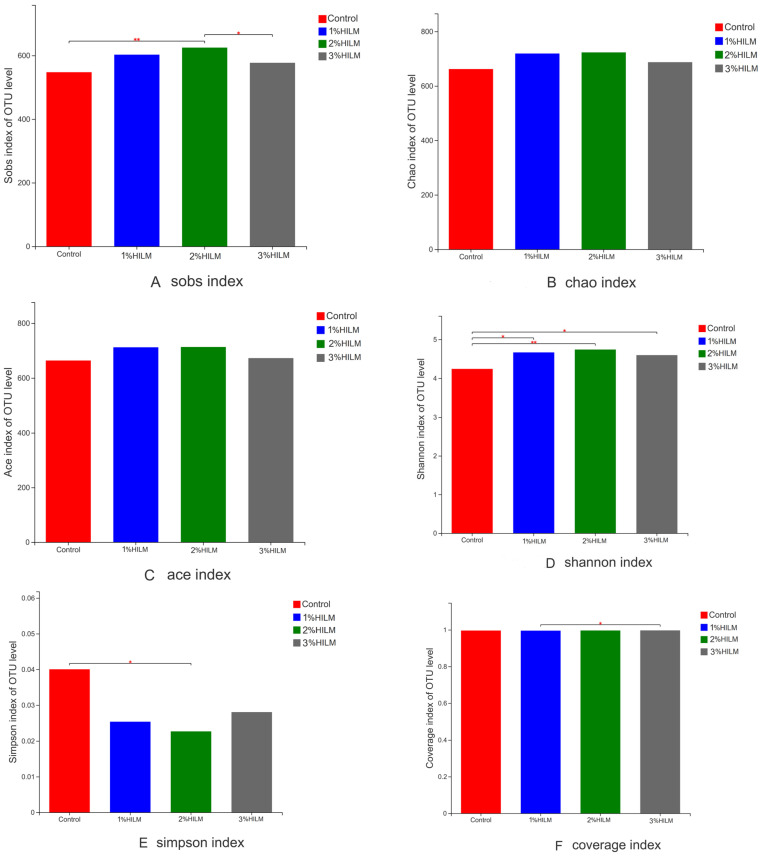
Alpha indexes data of caecum samples of hens. This figure shows the significant differences between these groups and makes two marks showing significant differences (0.01 < *p* ≤ 0.05 marked as *, 0.001 < *p* ≤ 0.01 marked as **). The abscissa is the group name, and the ordinate is the exponential average of each group. Control: based deit; 1% HILM: based deit + 1% *Hermetia illucens* larvae meal group; 2% HILM: based deit + 2% *Hermetia illucens* larvae meal group; 3% HILM: based deit + 3% *Hermetia illucens* larvae meal group.

Figure 1 shows large differences in community richness (as reflected by the Sobs, Chao, and ACE indices) or community diversity (as reflected by the Shannon and Simpson indices) in the cecum chyme at the OTU taxonomic level. The Sobs index of the 2% HILM group was significantly higher than that of the control group or 3% HILM group (*p* ≤ 0.05; Figure 1A). The Chao and ACE indices of the 2% HILM group were slightly higher than those of the other groups (*p* > 0.05; Figure 1B,C). The Shannon indices of the 1% HILM group 2% HILM group and 3% HILM group (*p* ≤ 0.05) were higher than the Shannon index of the control group, and the 2% HILM group had the highest Shannon index (Figure 1D). The Simpson index of the 2% HILM group was significantly lower than that of the control group (*p* ≤ 0.05; Figure 1E). The results showed that HILM supplementation increased the cecal microbial community richness and diversity of laying hens to a certain extent, and the 2% HILM group had the best effect. The coverage indices of each group (99.59%, 99.54%, 99.63%, and 99.68%) were higher than 99.5%, indicating that the cecum samples in each group met the requirements of sequencing and reflected the real situation of intestinal flora composition (Figure 1F).

#### 3.2.3. Circos Samples and Species Relationship Map

**Figure 2:**
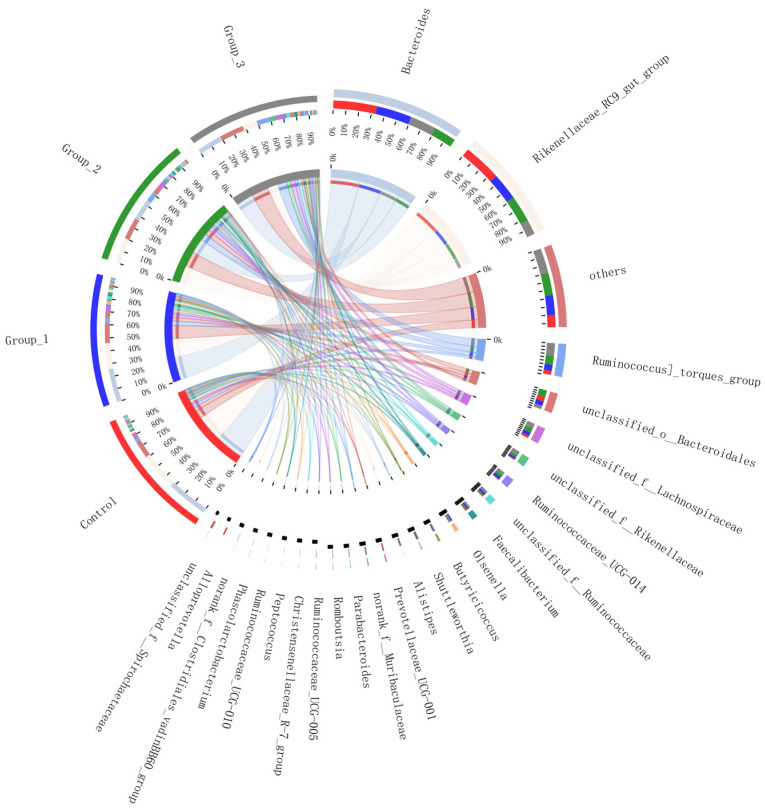
Circos Circos samples and species relationship map at the Genus level. In this Figure, the small semicircle (left semicircle) represents the species composition in the sample; the color of the outer ribbon represents the group from which it comes, the color of the inner ribbon represents the species, and the length represents the relative abundance of the species in the corresponding sample. The large semicircle (right semicircle) represents the distribution of species in different samples at the phylum level. The outer color band represents the species, the inner color band represents different groups, and the length represents the distribution proportion of the sample in a species. Control: based deit; 1% HILM: based deit + 1% *Hermetia illucens* larvae meal group; 2% HILM: based deit + 2% *Hermetia illucens* larvae meal group; 3% HILM: based deit + 3% *Hermetia illucens* larvae meal group.

The circos samples and species relationship map (Figure 2) reflects the distribution of dominant species in each group at the genus levels. At the genus level, 177 genera were detected through Illumina Miseq sequencing. *Bacteroides* and *Rikenellaceae_RC9_gut_group* were the most dominant genera in each group. The relative abundance rates of *Bacteroides* were 33.32%, 26.83%, 17.21%, and 20.06%, and the relative abundance rates of *Rikenellaceae_RC9_gut_group* were 29.59%, 20.66%, 20.62%, and 11.57% (Figure 2).

#### 3.2.4. Beta Diversity Analysis

PCoA analysis (based on Bray–Curtis distance algorithm) was conducted at the phylum, family, and genus levels, and ANOSIM (permutation_number: 999) was used to test the difference between groups for the calculation of the R and *p* values.

**Figure 3:**
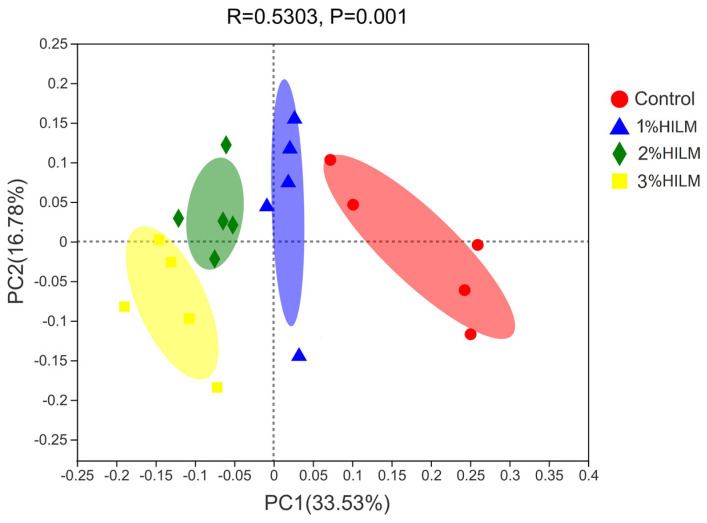
PCoA analysis at the Genus level. In this Figure, the *x*−axis and *y*−axis represent the two selected principal coordinate axes, and the percentage represents the explanatory value of the principal coordinate axis to the difference in sample composition. The scale of the *x*−axis and *y*−axis is relative distance, which has no practical significance. The closer the two sample points are, the more similar the species composition of the two samples. Control: based deit; 1% HILM: based deit + 1% *Hermetia illucens* larvae meal group; 2% HILM: based deit + 2% *Hermetia illucens* larvae meal group; 3% HILM: based deit + 3% *Hermetia illucens* larvae meal group.

Figure 3 shows the different clustering trends of each group at the genus levels, respectively. The samples of each group were significantly separated (*p* = 0.001) in the PCoA map, indicating significant differences in cecal community composition among the groups. The R value was near 1 at the genus level (statistic = 0.5303), indicating that the difference between groups was greater than that within groups and the grouping of the experiment was reasonable.

#### 3.2.5. Species Difference Analysis

At the phylum level, the relative abundance rates of *Bacteroidetes* and *Firmicutes* significantly changed (*p* < 0.001). The relative abundance rates of *Bacteroidetes* in the HILM-supplementation groups were significantly lower than relative abundance in the control group (*p* < 0.001), and the relative abundance rates of *Firmicutes* in the HILM-supplementation groups were significantly higher than relative abundance in the control group (*p* < 0.001; Table S1).

At an increased HILM, the relative abundance rates of *Ruminococcaceae*, *Lachnospiraceae*, *Erysipelotrichaceae*, *Peptostreptococcaceae*, and *Christensenellaceae* (all belonging to *Firmicutes*) increased (Table S2). Table S2 shows that the relative abundance rates of *Ruminococcaceae*, *Lachnospiraceae*, *Erysipelotrichaceae*, and *Christensenellaceae* in the 2% HILM and 3% HILM groups were significantly higher than those in the control group (*p* ≤ 0.05), and the relative abundance of *Peptostreptococcaceae* in the 2% HILM group was significantly higher than that in the control group (*p* ≤ 0.05). However, at an increased HILM, the relative abundance rates of *Rikenellaceae*, *Bacteroidaceae*, and *Prevotellaceae* (all belonging to *Bacteroidetes*) decreased (Table S2). Table S2 shows that the relative abundance rates of *Rikenellaceae* and *Bacteroidaceae* in the 3% HILM group were significantly lower than those in the control group (*p* ≤ 0.05), the relative abundance of *Bacteroidaceae* in the 2% HILM group was significantly lower than its relative abundance rates in the control and 1% HILM groups (*p* ≤ 0.05), and the relative abundance of *Prevotellaceae* in the HILM-supplementation groups was significantly lower than that in the control group (*p* ≤ 0.05).

**Figure 4:**
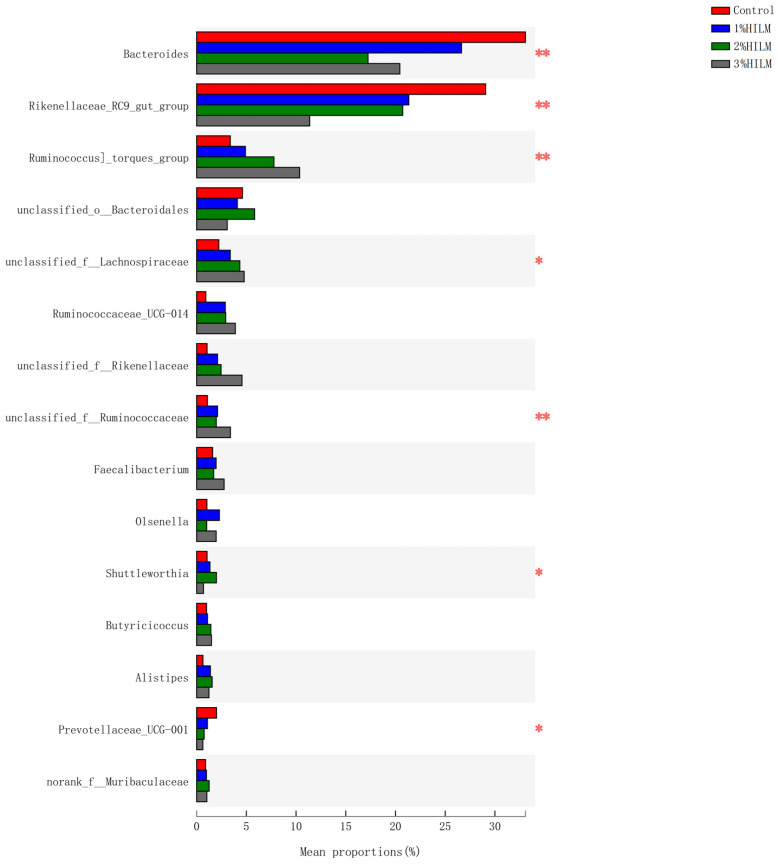
Significant difference test between groups at the Genus level. In this Figure, the *y*-axis represents the species name at the phylum level, the *x*-axis represents the average relative abundance in different groups of species, and the columns with different colors represent different groups; * means the value of *p*, * 0.01 < *p* ≤ 0.05, ** 0.001 < *p* ≤ 0.01; Control: based deit; 1% HILM: based deit + 1% *Hermetia illucens* larvae meal group; 2% HILM: based deit + 2% *Hermetia illucens* larvae meal group; 3% HILM: based deit + 3% *Hermetia illucens* larvae meal group.

Further analysis at the genus level can clearly reflect the trends of changes in the cecal flora structures in different treatment groups. Figure 4 shows the genus with an average relative abundance in the top 15, and significant differences between groups were tested through one-way ANOVA analysis. Table S3 shows the genus with significant differences (*p* ≤ 0.05) and an abundance of >0.02%.

At an increased HILM, the relative abundance rates of *Bacteroides*, *Rikenellaceae_RC9_gut_group*, *Prevotellaceae_UCG-001*, *Alloprevotella*, *Sphaerochaeta*, and *unclassified_f__Prevotellaceae* (all belonging to *Bacteroidetes*) decreased (*p* ≤ 0.05; Table S3). However, the relative abundance rates of the *[Ruminococcus]_torques_group*, *Shuttleworthia*, *Lactobacillus*, *Romboutsia*, *Christensenellaceae_R-7_group*, *Ruminococcaceae_UCG-010*, *Ruminococcaceae_UCG-013*, *Turicibacter*, *unclassified_f__Lachnospiraceae*, *unclassified_f__Ruminococcaceae*, *norank_f__Clostridiales_vadinBB60_group*, *norank_f__Ruminococcaceae*, *Family_XIII_AD3011_group*, and *Eubacterium]_nodatum_group* and *unclassified_f__Eggerthellaceae* (all belonging to *Firmicutes*) increased with HILM (Figure 4). Interestingly, all the relative abundance rates of *Faecalibacterium*, *Alistipes*, and *Butyricicoccus* (all butyric-acid-producing bacteria) in the HILM-supplementation groups were higher than those in the control group (*p* > 0.05; Figure 4).

## 4. Discussion

### 4.1. Effect of Hermetia illucens Larvae Meal on the Production Performance of Laying Hens

Some studies showed that dietary supplementation with HILM had a significantly positive impact on production performance [27,49,50]. Bovera et al. (2018) [49] pointed out that a diet containing 7.3% partially defatted *H. illucens* larvae significantly improved laying rate but had no significant effect on the feed intake and feed conversion rate of laying hens (from 16 to 40 weeks old, “Hy-line Brown”; *p* > 0.05). Widjastuti et al. (2014) [50] pointed out that dietary supplementation with 2.5%, 5.0%, 7.5%, and 10.0% HILM significantly increased the laying and feed conversion rates of egg quails aged from 6 to 20 weeks (*p* ≤ 0.05). Our current research showed that the laying rate linearly increased (*p* ≤ 0.05) and feed/egg and cracked-egg rate linearly decreased (*p* ≤ 0.05) with increasing HILM levels (*p* ≤ 0.05), but feed intake and egg weight had no effect. The results of these studies were inconsistent, possibly because different poultry breeds at different age stages were used, with different processing methods, and HILM contents. Currently, the specific reason and mechanism by which HILM regulates the production performance of laying hens is unclear; thus, explaining the differences between feed treatments is difficult. Therefore, this study further explored the effect of HILM on laying hens on the basis of the microbial flora.

### 4.2. Effect of Hermetia illucens Larvae Meal on the Cecal Microflora of Laying Hens

Microflora is affected by many factors. Dietary composition is the main factor affecting the microflora [51], and feed source and local feed improvements significantly affect intestinal microbial community [52]. The use of HILM as a feed component has a significant impact on the intestinal microflora of livestock and poultry [34,53,54].

The rarefaction curves showed that the sequencing sample size was sufficient, and the coverage index (all more than 99.5%) showed that the samples in each group met the requirements of sequencing. Hence, the sequencing results were able to reflect the real situation of intestinal flora composition. Alpha diversity analysis showed that dietary HILM supplementation increased the cecal community richness (Sobs, Chao, and Ace indices) and community diversity (Shannon and Simpson indices) of laying hens at the late laying period. The 2% HILM group had the highest cecum community richness and community diversity. This result indicated that dietary HILM supplementation can increase cecal microflora diversity in laying hens. The results of the present study are similar to the results of Kawasaki et al. (2019) [11], who showed that dietary supplementation with 10% *H. illucens* prepupa meal significantly increased cecal microflora diversity in laying hens (from 55 to 59 weeks old; “Julia”).

The microflora in the chicken cecum is mainly composed of nine phyla. Approximately 98% of the bacteria can be classified into four phyla: *Bacteroidetes*, *Firmicutes*, *Proteobacteria*, and *Actinobacteria*. *Firmicutes* and *Bacteroidetes* are the dominant flora [55,56,57]. Yan et al. (2017) [58] pointed out that *Bacteroidetes* (relative abundance > 50%) and *Firmicutes* (relative abundance = 26%) were the dominant bacteria at the phylum level, and *Bacteroides* (relative abundance range: from 21.7% to 23.6%) and *Prevotella* (relative abundance range: from 3.9% to 6.2%) were the dominant bacteria at the family level in the ceca of 60-week-old brown-egg dwarf layers. In this study, the dominant bacteria in each group were *Bacteroidetes* (relative abundance: from 46.26% to 76.78%) and *Firmicutes* (relative abundance: from 19.17% to 45.52%) at the phylum level, and the dominant bacteria in each group all were *Bacteroides* (relative abundance: from 17.21% to 33.32%) and *Rikenellaceae_RC9_gut_group* (relative abundance: from 11.57% to 29.59%) at the family level. At the phylum level, the results of this experiment (control group) and Yan et al. (2017) [58] all showed that *Bacteroidetes* and *Firmicutes* were the most dominant flora in the ceca of laying hens at the late laying period, and *Bacteroidetes* had the absolute advantage. Callaway et al. (2009) [59] showed that *Bacteroidetes* occupied two-thirds of the cecal microflora in the ceca of laying hens (from 75 to 80 weeks old; “Single Comb White Leghorn”). At the genus level, Yan et al. (2017) [58] obtained different results, possibly because they used a different dietary composition, age stage, and laying hen breed. Dietary composition and animal breed are the main reasons for differences in cecal microbiota composition [51,52,60], and the cecal microflora of chicken continues to evolve with increasing age [61,62]. However, the relative abundance of two phyla (*Bacteroidetes* and *Firmicutes*) were basically the same, and each phylum accounted for approximately 40–45% of the intestinal flora in the ceca of ISA Brown laying hens aged 7 months or older (typical stage of full laying adult layers) [62]. In the present study, the relative abundance rates of *Bacteroidetes* and *Firmicutes* in the ceca of the laying hens significantly changed with increasing HILM, especially *Bacteroidetes* (average relative abundance was 45.95%) and *Firmicutes* (average relative abundance was 45.88%) in the 3% HILM group. These changes seemed to show positivie trends. At the late laying period, laying hens are in the period of laying fatigue, and body resistance is reduced and easily disturbed by bacteria. The flora of *Firmicutes* were dominant in the intestines of young and growing chickens and produced substantial amounts of butyric acid [63], which reduced pH and inhibited the growth of acid-sensitive pathogens [64,65], improved mineral absorption [66], and supplied growing intestinal cells and intestine-associated lymphoid tissues [67,68]. Butyric acid producers are mainly found in *Firmicutes* and include *Faecalibacterium*, *Roseburia*, and *Eubacterium* [69]. In the present study, *Faecalibacterium*, *Eubacterium]_nodatum_group*, and *Butyricicoccus* were detected at the genus level. The relative abundance rates of *Eubacterium]_nodatum_group* in the 2% and 3% HILM groups were significantly higher than those in the control group. The relative abundance rates of *Faecalibacterium* and *Butyricicoccus* in the HILM-supplementation groups were higher than those in the control group. The relative abundance rates of *Faecalibacterium* and *Butyricicoccus* in the 3% HILM group were the highest. A possible reason for the significant increase in the abundance of *Firmicutes* was that the relative abundance rates of *Faecalibacterium*, *Eubacterium]_nodatum_group*, and *Butyricicoccus* in the 3% HILM group considerably increased. This result indicated that HILM affected the production of intestinal short-chain fatty acids (such as butyric acid) to a certain extent. Dietary supplementation with 14.6% HILM significantly increased the amounts of butyrate, acetate, and total volatile fatty acids (VFAs) in ceca of 16-to-40-week-old Hy-line Brown laying hens [29]. and Borrelli et al. (2017) [29] and Cutrignelli et al.(2018) [70] obtained similar results.

ANOSIM based on Bray–Curtis distance showed that the differences among groups were significantly greater than differences within groups, indicating that grouping was significant. The results of PCoA analysis showed significant differences in the relative abundance rates of cecal flora of the groups during the late laying period. At the phylum level, the relative abundance of *Firmicutes* increased, whereas that of *Bacteroidetes* decreased in the HILM addition groups. Borrelli et al. (2017) [53] obtained similar results and pointed out that the use of defatted HILM as a protein source significantly affects the species composition and relative abundance of cecal microflora of laying hens (from 24 to 45 weeks old, “Lohmann Brown classic”). After conducting a species composition analysis (bar chart) and PCoA analysis, they found that the relative abundance of *Bacteroidetes* decreased (control group: 31.52% ± 3.28%; HI group: 25.40% ± 1.30%); the relative abundance of *Firmicutes* increased (control group: 49.28% ± 3.16%; *Hermetia illucens* (HI) group: 57.69% ± 2.37%). An increase in dietary fiber content leads to a significant difference in the cecal microbial community [71]. In the present study, dietary supplementation with 2% and 3% HILM increased the relative abundance rates of fiber-degrading bacteria (such as *Ruminococcaceae* and *Lachnospiraceae*) and decreased the relative abundance rates of opportunistic pathogens (such as *Peptostreptococcaceae*) [72]. These effects may be related to chitin in HILM. Chitin is a cellopolysaccharide [73] usually found in insects, crustaceans, bacteria, fungi, and other low organisms and is the only alkaline polysaccharide with positive ions in nature. A low chitin level can restore intestine microbial community balance in humans [74] or mice [75]. The percentages of chitin in the larvae, prepupae, pupae, and adults of *H. illucens* are approximately 3.6%, 3.1%, 14.1%, and 2.9%, respectively, and chitin from different stages of *H. illucens* was α-chitin, with a similar thermal stability [76,77]. Previous studies reported that the percentages of chitin in HILM are 4.97% [78], 4.65% [35], and 6.43% [21]. Insect chitin may be decomposed into chitosan and chito-oligosccharide by acid chitinase in the glandular stomach and intestines of chickens [79], and chitin may enter the large intestine and be used by microorganisms as a fermentable substrate [80]. Borrell et al. (2017) [53] pointed out that *Alkaliophus Transvaalensis*, *Christensenella minuta* (belonging to the family *Christensenellaceae*), and *Flavonifractor plautii* (belonging to the family *Ruminococcaceae*) potentially degrade chitin in HILM. In our study, the relative abundance rates of *Ruminococcaceae* and *Christensenellaceae* significantly increased with HILM content, indicating that HILM increased the amount of *C. minuta* and *F. plautii* and then promoted the degradation of chitin and produced chitosan and chito-oligosccharide. Chitosan can significantly improve the fecal microflora [81] and significantly increase the proportions of *Lactobacillus* and *Escherichia coli*. In addition, Yu et al. (2019) [34] studies showed that a dietary addition of 4% HILM significantly increased the relative abundance rates of *Lactobacillus*, *Oribacterium*, and *Faecalibacterium* in the colons of finishing pigs. In the present study, dietary HILM supplementation slightly increased the relative abundance rates of *Faecalibacterium* and significantly increased the relative abundance rates of *Lactobacillus* and *unclassified_f__Lachnospiraceae*. Therefore, chitin and its derivatives (as a potential probiotics) in the diets of laying hens fed with HILM may cause significant changes in the cecal microflora. However, the exact mechanism by which HILM affects the cecal microflora of laying hens remains unclear.

## 5. Conclusions

Under the conditions of this experiment, dietary supplementation with HILM improved the laying rate and reduced the cracked egg rate of laying hens at the late laying period on the production performance. On the cecal microflora, dietary supplementation with HILM improved the relative abundance rates of *Firmicutes* and *Bacteroidetes* at the phylum level and the relative abundance rates of *Ruminococcaceae*, *Lachnospiraceae*, *Erysipelotrichaceae*, *Peptostreptococcaceae Christensenellaceae Rikenellaceae Bacteroidaceae* and *Prevotellaceae* at the family level, and thus improved the microbial structure in the cecal of laying hens.

## Figures and Tables

**Table 1:** The chemical components of *Hermetia illucens* larvae meal (HILM) (dry matter) (%).

Items	*Hermetia illucens* Larvae Meal
DM	90.02
CP	37.60
ME/(MJ/kg)	8.74
EE	36.00
crude ash	6.20
Met	0.69
Lys	2.18
Ca	0.96
P	0.83

Abbreviations: DM, dry matter, ME, metabolizable energy; CP, crude protein; EE, ether extract; Met, Methionine; Lys, lysine; Ca, calcium; P, phosphorous; dry matter and ether extract were measured values, while the other nutrients had calculated values.

**Table 2:** Composition and nutrient levels of the diets (air-dry basis) (%).

Items	Control	1% HILM	2% HILM	3% HILM
Ingredients				
Corn	62.00	61.63	61.25	60.88
Soybean meal	24.00	23.37	22.75	22.12
Wheat bran	2.00	2.00	2.00	2.00
Limestone	8.00	8.00	8.00	8.00
Soybean oil	1.00	1.00	1.00	1.00
*Hermetia illucens* larva meal	0.00	1.00	2.00	3.00
Premix *	3.00	3.00	3.00	3.00
Total	100.00	100.00	100.00	100.00
Nutrient levels ^&^				
ME/(MJ/kg)	11.39	11.39	11.40	11.40
DM	89.86	89.67	89.78	89.84
CP	15.80	15.81	15.85	15.89
EE	3.36	3.70	4.05	4.40
Met	0.25	0.26	0.26	0.27
Lys	0.76	0.78	0.79	0.80
Trp	0.20	0.19	0.23	0.24
Phe	0.58	0.60	0.63	0.64
Thr	0.62	0.64	0.65	0.68
Ile	0.72	0.74	0.75	0.77
Leu	1.07	1.10	1.13	1.4
Val	0.62	0.62	0.63	0.65
Na	0.3	0.3	0.3	0.3
Ca	3.50	3.5	3.5	3.5
AP	0.34	0.34	0.34	0.34
TP	0.50	0.5	0.5	0.5

Abbreviations: DM, dry matter, ME, metabolizable energy; CP, crude protein; EE, ether extract; Met, Methionine; Lys, lysine; Trp, tryptophan; Phe, phenylalanine; Thr, threonine; Ile, isoleucine; Leu, leucine; Val, valine; Ca, calcium; AP, available phosphorous; TP, total phosphorous; * Provide per kg of diet: vitamin A 12,000 IU, vitamin D3 2700 IU, vitamin E 26 IU, vitamin K 1.8 mg, vitamin B1 2.20 mg, vitamin B2 8 mg, vitamin B6 2.0 mg, vitamin B12 0.03 mg, nicotinic acid 36.0 mg, D-pantothenate 8 mg, folic acid 1.2 mg, biotin 0.10 mg, choline chloride 100 mg, Ca 15 g, P 5 g, Mn 75 mg, Fe 90 mg, Cu 7 mg, Zn 75 mg, DL-Met 0.2%, NaCl 0.3%. ^&^ Dry matter and ether extract were measured values, while the other nutrients were calculated values.

**Table 3:** Effects of *Hermetia illucens* larvae meal on production performance of laying hens.

Items	Control ^#^	1% HILM ^#^	2% HILM ^#^	3% HILM ^#^	SEM *	*p*-Value		
						Overall	Linear	Quadratic
Egg laying rate/(%)	85.36 ^c^	85.95 ^bc^	86.52 ^ab^	87.51 ^a^	1.02	0.018	0.037	0.54
Feed/egg	2.22 ^a^	2.18 ^ab^	2.13 ^bc^	2.10 ^c^	0.08	0.041	0.032	0.306
cracked-egg rate/(%)	0.68 ^a^	0.54 ^ab^	0.46 ^ab^	0.39 ^b^	0.79	0.024	0.041	0.114
Average egg weight/(g)	67.17	67.10	66.93	66.98	0.42	0.616	0.379	0.787
ADFI/(g)	126.73	126.79	123.67	125.41	3.68	0.531	0.756	0.306

In the same row, values with no or the same letter superscripts mean no significant difference (*p* > 0.05), while those with different small letter superscripts mean significant difference (*p* ≤ 0.05). ^#^ Control:based deit; 1% HILM: based deit + 1% *Hermetia illucens* larvae meal group; 2% HILM: based deit + 2% *Hermetia illucens* larvae meal group; 3% HILM: based deit + 3% *Hermetia illucens* larvae meal group. * means of n = 5 observations. ^a,b,c^ Means within a row with no common superscripts differ significantly (*p* ≤ 0.05).

## Data Availability

The data presented in this study are available on request from the corresponding author.

## Supplementary Materials

The following supporting information can be downloaded at: https://www.mdpi.com/article/10.3390/vetsci10050364/s1, Figure S1: Rarefaction curves. Table S1: One-way ANOVA test data at the Phylum level /% *. Table S2: One-way ANOVA test data at the Family level /% *. Table S3: One-way ANOVA test data at the Genus level /% *.
