# Supplementary material for: Effects of Black Soldier Fly Larvae (Hermetia illucens Larvae) Meal on the Production Performance and Cecal Microbiota of Hens
Source: Vet Sci. 2023 May 19;10(5):364. doi: 10.3390/vetsci10050364 (PMC10222152; doi:10.3390/vetsci10050364)
Supplement: Supplementary file 1 [file vetsci-10-00364-s001.zip › vetsci-2164391-supplementary.pdf]

Supplementary Materials:

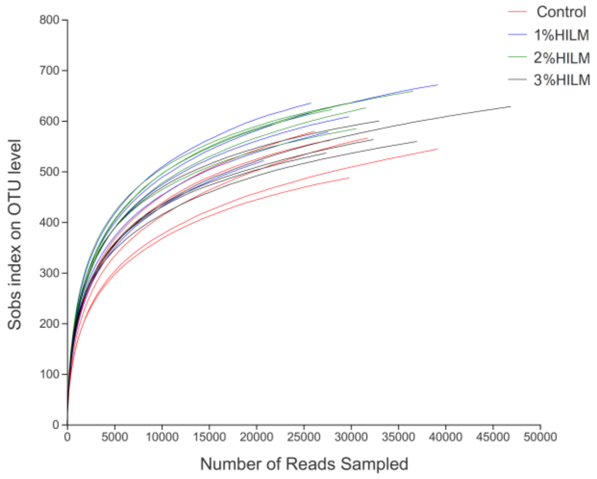

Figure S1. Rarefaction curves. Abscissa represents the amount of sequencing data randomly selected; Ordinate represents observed species number (sobs index). Control:based deit; 1% HILM：based deit + 1% *Hermetia illucens* larvae meal group; 2 % HILM：based deit + 2% *Hermetia illucens* larvae meal group; 3% HILM：based deit + 3% *Hermetia illucens* larvae meal group.

Table S1. One-way ANOVA test data at the Phylum level /% *.

Species Name	Control #	1% HILM #	2% HILM #	3% HILM #	*p*-Value
Bacteroidetes	76.460 ± 5.209 [a]	62.060 ± 1.716 [b]	53.880 ± 1.996 [c]	45.950 ± 2.636 [d]	<0.001
Firmicutes	19.420 ± 4.606 [c]	31.750 ± 1.852 [b]	41.910 ± 1.720 [a]	45.880 ± 2.632 [a]	<0.001
Actinobacteria	1.559 ± 0.649	3.190 ± 1.851	1.723 ± 0.823	3.708 ± 2.093	0.1626
Proteobacteria	0.343 ± 0.194	0.800 ± 0.560	0.814 ± 0.464	1.579 ± 1.980	0.1558
Spirochaetes	1.469 ± 1.125	0.285 ± 0.169	0.344 ± 0.152	0.605 ± 0.762	0.2306
Patescibacteria	0.163 ± 0.141	0.521 ± 0.437	0.150 ± 0.149	0.975 ± 1.172	0.2608
Tenericutes	0.177 ± 0.150	0.326 ± 0.126	0.524 ± 0.285	0.442 ± 0.304	0.1586
Synergistetes	0.087 ± 0.019	0.309 ± 0.158	0.109 ± 0.072	0.374 ± 0.553	0.0926
Epsilonbacteraeota	0.068 ± 0.026	0.181 ± 0.160	0.105 ± 0.070	0.299 ± 0.177	0.0910
Deferribacteres	0.082 ± 0.084	0.201 ± 0.129	0.177 ± 0.190	0.095 ± 0.084	0.3988
WPS-2	0.077 ± 0.094	0.089 ± 0.117	0.145 ± 0.201	0.052 ± 0.090	0.8326
unclassified_k__norank_d__ Bacteria	0.034 ± 0.039	0.111 ± 0.211	0.032 ± 0.033	0.012 ± 0.009	0.4005
Verrucomicrobia	0.021 ± 0.040	0.089 ± 0.180	0.007 ± 0.007	0.010 ± 0.015	0.6911
Elusimicrobia	0.026 ± 0.038	0.029 ± 0.013	0.054 ± 0.062	0.012 ± 0.024	0.4924
Cyanobacteria	0.012 ± 0.008	0.046 ± 0.051	0.012 ± 0.012	0.009 ± 0.013	0.5744

* This table shows the Phylum with average relative abundance in the top 15; datas were "Mean ± SEM";. # Control:based deit; 1% HILM: based deit + 1% *Hermetia illucens* larvae meal group; 2% HILM: based deit + 2% *Hermetia illucens* larvae meal group; 3% HILM: based deit + 3% *Hermetia illucens* larvae meal group. a, b, c. Means not sharing the same superscripts in a row differ significantly ($p \leq 0.05$).

Table S2. One-way ANOVA test data at the Family level /% *.

Species Name	Control #	1% HILM #	2% HILM #	3% HILM #	*p*-Value
Rikenellaceae	30.750 ± 8.415 a	24.830 ± 3.922 ab	24.750 ± 3.556 ab	17.170 ± 3.582 b	0.0195
Bacteroidaceae	33.070 ± 5.459 a	26.640 ± 4.033 ab	17.240 ± 1.189 cd	20.440 ± 5.213 bd	0.0010
Ruminococcaceae	8.257 ± 2.330 b	14.220 ± 3.103 ab	16.550 ± 3.909 a	20.190 ± 6.248 a	0.0054
Lachnospiraceae	7.521 ± 2.392 cd	11.230 ± 1.761 bc	15.830 ± 3.731 ab	17.890 ± 4.032 a	0.0043
unclassified_o__Bacteroidales	4.609 ± 2.170	4.093 ± 1.144	5.831 ± 2.009	3.077 ± 0.535	0.0696
Prevotellaceae	4.842 ± 1.066 a	2.274 ± 1.175 b	1.418 ± 0.767 b	1.534 ± 0.986 b	0.0025
Atopobiaceae	1.037 ± 0.509	2.303 ± 1.391	1.015 ± 0.649	1.977 ± 1.087	0.2046
Tannerellaceae	0.752 ± 0.401	1.586 ± 0.783	1.529 ± 0.722	0.944 ± 0.304	0.1655
Peptococcaceae	0.849 ± 0.447	1.225 ± 0.760	1.269 ± 0.372	0.998 ± 0.260	0.4690
Erysipelotrichaceae	0.424 ± 0.214 b	0.580 ± 0.329 b	1.851 ± 0.629 a	1.378 ± 0.460 a	0.0034
Muribaculaceae	0.901 ± 0.688	0.990 ± 0.450	1.257 ± 0.966	1.029 ± 1.103	0.9378
Peptostreptococcaceae	0.258 ± 0.326 cd	0.324 ± 0.288 cd	2.035 ± 1.057 ab	1.230 ± 0.877 bd	0.0280
Christensenellaceae	0.349 ± 0.128c	0.726 ± 0.369 bc	1.268 ± 0.273 ab	1.312 ± 0.721 ab	0.0012
Acidaminococcaceae	0.867 ± 0.709	1.697 ± 1.786	0.287 ± 0.242	0.594 ± 0.616	0.2263
Barnesiellaceae	0.501 ± 0.396	0.815 ± 0.662	1.026 ± 0.590	1.015 ± 0.680	0.3918

* This table shows the Famly with average relative abundance in the top 15; datas were "Mean ± SEM"; # Control:based deit; 1% HILM: based deit + 1% *Hermetia illucens* larvae meal group; 2% HILM: based deit + 2% *Hermetia illucens* larvae meal group; 3% HILM: based deit + 3% *Hermetia illucens* larvae meal group.a, b,c Means not sharing the same superscripts in a row differ significantly ($p \leq 0.05$).

Table S3. One-way ANOVA test data at the Genus level /% *.

Species Name	Control #	1% HILM #	2% HILM #	3% HILM #	*p*-Value
Bacteroides	33.070 ± 5.459 ab	26.640 ± 4.033 bc	17.240 ± 1.189 d	20.440 ± 5.213 c	0.0010
Rikenellaceae_RC9_gut_group	29.070 ± 9.097 a	21.340 ± 4.238 a	20.730 ± 3.580 ab	11.370 ± 2.419 b	0.0016
Ruminococcus]_torques_group	3.388 ± 1.029 cd	4.906 ± 2.187 bd	7.777 ± 2.185a b	10.360 ± 2.792 a	0.0030
unclassified_f__Lachnospiraceae	2.229 ± 0.822 b	3.382 ± 0.779 ab	4.346 ± 1.486 a	4.781 ± 1.225 a	0.0235
unclassified_f__Ruminococcaceae	1.088 ± 0.126 b	2.106 ± 0.655 b	1.977 ± 0.544 b	3.402 ± 0.815 a	0.0013
Shuttleworthia	1.049 ± 0.574 ab	1.342 ± 0.597 ab	1.991 ± 0.697 a	0.690 ± 0.223 b	0.0233
Prevotellaceae_UCG-001	2.001 ± 0.618 a	1.085 ± 0.241 b	0.757 ± 0.371 b	0.627 ± 0.375 b	0.0172
Romboutsia	0.250 ± 0.319 b	0.285 ± 0.273 b	1.948 ± 1.039 a	1.204 ± 0.844 ab	0.0286
Christensenellaceae_R-7_group	0.337 ± 0.128 b	0.701 ± 0.354 ab	1.231 ± 0.271 a	1.279 ± 0.720 a	0.0014
Ruminococcaceae_UCG-010	0.322 ± 0.160 b	0.538 ± 0.266 b	1.406 ± 0.407 a	1.147 ± 0.244 a	0.0006
norank_f__Clostridiales_vadinBB60_group	0.409 ± 0.206 b	0.856 ± 0.128 ab	1.248 ± 0.547 a	0.639 ± 0.565 ab	0.0184
Alloprevotella	1.531 ± 0.939 a	0.158 ± 0.147 b	0.074 ± 0.038 b	0.287 ± 0.178 b	0.0276
Ruminococcaceae_UCG-013	0.209 ± 0.140 b	0.437 ± 0.311 ab	0.676 ± 0.208 a	0.268 ± 0.148 b	0.0214
Turicibacter	0.141 ± 0.129 b	0.081 ± 0.082 b	0.883 ± 0.368 a	0.404 ± 0.254 b	0.0083
Lactobacillus	0.108 ± 0.070	0.326 ± 0.311	0.296 ± 0.094	0.284 ± 0.180	0.0391
norank_f__Ruminococcaceae	0.087 ± 0.031 b	0.333 ± 0.215 a	0.187 ± 0.053 ab	0.387 ± 0.108 a	0.0017
Sphaerochaeta	0.402 ± 0.202 a	0.261 ± 0.178 ab	0.174 ± 0.112 ab	0.043 ± 0.028 b	0.0124
Family_XIII_AD3011_group	0.053 ± 0.019 b	0.108 ± 0.048 b	0.240 ± 0.108 a	0.166 ± 0.064 ab	0.0074
unclassified_f__Prevotellaceae	0.238 ± 0.091 a	0.152 ± 0.064 ab	0.079 ± 0.059 b	0.065 ± 0.053 b	0.0284
unclassified_f__Eggerthellaceae	0.035 ± 0.019	0.090 ± 0.014	0.073 ± 0.030	0.164 ± 0.144	0.0074
Eubacterium]_nodatum_group	0.026 ± 0.012 b	0.052 ± 0.016 b	0.097 ± 0.032 a	0.108 ± 0.016 a	0.0001

* This table shows the genus with significant difference ($p \leq 0.05$) and abundance > 0.02%; datas were "Mean ± SEM"; # Control:based deit; 1% HILM: based deit + 1% *Hermetia illucens* larvae meal group; 2% HILM: based deit + 2% *Hermetia illucens* larvae meal group; 3% HILM: based deit + 3% *Hermetia illucens* larvae meal group. a, b,c Means not sharing the same superscripts in a row differ significantly ($p \leq 0.05$).
